# Validation of a photoelectric sensor system to detect oviposition timing in individually caged broiler breeder hens

**DOI:** 10.1016/j.psj.2025.105932

**Published:** 2025-10-01

**Authors:** L.R. Sroda, J.D. Davis, K. Diehl, C.M. Edge, M.R. Berger, K.E.C. Elliott, B.R. Flack, C.L. Hanlon

**Affiliations:** a National Poultry Technology Center, Auburn University, Auburn, AL, USA; b Animal Biosciences & Biotechnology Laboratory, USDA ARS, Beltsville, MD, USA; c Poultry Science, Auburn University, Auburn, AL, USA; d Poultry Research Unit, USDA ARS, Mississippi State, MS, USA; e Genetics, Cobb-Vantress, Siloam Springs., AR, USA

**Keywords:** Precision animal management, Oviposition, Broiler breeder, Photoelectric sensor

## Abstract

Understanding the time of oviposition in broiler breeders is an important metric for improving precision animal and feeding management in the U.S. The objective of this project was to validate the use of a system using photoelectric sensors (PES) to detect the time of oviposition of individually caged broiler breeders. The system was validated using 101 broiler breeder hens housed in individual cages within an environmentally controlled facility over a two-week period. The detection system consisted of a single PES and opposing reflector installed on 3D-printed dividers that separated the egg saver for each cage. The PESs were measured with datalogging systems using a 1-min sampling rate. A camera system and staff records were used to validate the PES system's accuracy. The PES system accurately detected 94.7 % (1339/1414) of events within 3 min of the camera observation. Addressing correctable errors caused by cage design issues could improve the accuracy of the system to 98.7 % (1396/1414). Current limitations of the PES system include the inability to detect soft-shelled or cracked eggs that do not roll into the egg saver, the inability to distinguish when a hen has laid two eggs in the egg saver for a collection period, and the requirement to deactivate the system during the dark period to prevent photorefractoriness of broiler breeders by the red light emitted from the sensors. Determining the time of oviposition was streamlined and efficient using a PES system, reducing data acquisition time (< 0.2 h) compared to a camera-based system (>100 h). At an estimated cost of $369 per cage, the PES system offers a reliable, high-precision tool to explore any poultry egg production research where non-invasive identification of oviposition timing could provide insight into reproductive capacity and success.

## Introduction

Over the past decade, combined fertility and hatchability rates in broiler breeders have declined from 85 % to 79 %, posing a significant challenge for the poultry industry in meeting the growing global demand for broiler chicks. With losses estimated at up to 2.5 million unhatched eggs annually, concerns surrounding fertilization and egg quality have become increasingly prominent (National Agricultural Statistics Service, 2023). These outcomes are influenced, in part, by management practices that affect the timing of egg formation and the resulting time of lay, or oviposition.

While oviposition is the physical expulsion of the egg, these events typically occur in sequences, or clutches, consisting of one or more consecutive days of egg laying, followed by a pause day, during which no oviposition occurs (Fraps, 1955; Etches, 1984). The occurrence of oviposition relies on the successful completion of the ovulatory cycle, which is tightly regulated by endocrine signals. Follicle-Stimulating Hormone (FSH) facilitates follicle recruitment and early development (Palmer and Bahr, 1992), while the preovulatory surge of luteinizing hormone (LH) triggers ovulation of the largest (F1) follicle (Robinson et al., 1988; Yang et al., 1997; Robinson and Renema, 2003). This hormonal coordination maintains the follicular hierarchy, allowing the next follicle in line to progress toward ovulation. According to Lewis et al. (2004a) the complete ovulatory cycle takes ∼35 h, from the initial LH surge to oviposition. Thus, improved understanding of the precise timing of oviposition could allow for the development of non-invasive hormonal prediction models to optimize reproductive management.

The timing of oviposition events can be influenced by several factors, including genetic strain (Lewis et al., 2004a; Tůmova and Gous, 2012), lighting schedules (Lewis et al., 2001, 2004b), and feeding time (Backhouse and Gous, 2005), all of which can ultimately impact the internal and external components of the egg. In broiler breeders, most eggs are laid in the morning at peak production (Zakaria et al., 2005). However, as the flock ages, oviposition times tend to become more variable (Zakaria et al., 2005), expanding the time frame in which eggs must be collected. Since broiler breeders are typically fed restricted allocations each morning, this shift in oviposition timing may influence nutrient availability during critical stages of egg formation. However, this relationship remains unexplored.

Although hourly egg collection studies have reported no differences in fertility based on the time of oviposition (Novo et al., 1997; Elibol et al., 2002; Zakaria et al., 2005, 2009), some research suggests that first-of-sequence eggs may be associated with reduced fertility in broiler breeders (Goerzen et al., 1996) and turkey hens (Bacon and Nestor, 1979) undergoing artificial insemination. These findings highlight the importance of considering the time of oviposition and the sequence number of the egg laid when evaluating reproductive outcomes.

Despite the absence of clear differences in fertility rates directly associated with the time of oviposition, the timing of this event still plays a critical role in fertilization success. When hens are inseminated during the final hours of egg formation, fertility rates decline significantly. This decline is attributed to the presence of hard-shelled eggs in the oviduct, impeding sperm storage and reducing the likelihood of successful fertilization (Moore and Byerly, 1942; Malmstrom, 1943; Bornstein et al., 1960; Giesen et al., 1980). In the turkey industry, which relies solely on artificial insemination, improved knowledge of oviposition timing could help reduce the frequency of inseminations during suboptimal periods. Similarly, in naturally mated broiler breeder flocks, which often display increased mating activity during the afternoon (Moyle et al., 2010), aligning management strategies with oviposition timing may help minimize overlap between mating and egg-laying, enhancing reproductive efficiency.

Ultimately, noninvasive monitoring of oviposition offers the opportunity to track ovulation and eggshell formation rates on a daily basis. This would enhance our understanding of the relationship between feeding management and reproductive processes of broiler breeders, ultimately improving the cumulative production of settable eggs. Such insights are essential for optimizing precision feeding strategies and improving hatchability outcomes, ensuring the U.S. poultry industry can sustainably and efficiently meet production demands.

Despite the critical role that oviposition timing plays in fertility and egg quality, many studies have relied on manual hourly monitoring of battery cages (Giesen et al., 1980; Fasenko et al., 1992; Lewis et al., 2004a; Zakaria et al., 2005, 2009; Akil and Zakaria, 2015), dating back to Warren and Scott (1936). This process is labor-intensive, reliant on human observation, and lacks precision. This creates a significant barrier to understanding the dynamic interplay between management practices, reproductive physiology, and fertility and hatchability outcomes. With the growing need for reliable, noninvasive tools capable of continuously and accurately monitoring oviposition events in real time, researchers have tested various systems over the past decades. This has included a variety of physical mechanisms like a spring-hinged lever (Hinds, 1949) or stylus (Weiss and Sturkie, 1952); Bastian and Zarrow, 1954) that “tripped” when the egg rolled into the egg saver and recorded on a drum kymograph. While these systems were able to record oviposition time successfully, challenges remained regarding the optimal slope required for cage floors to ensure accurate detection, as well as potential interference from adjacent cages that may compromise reliability. Others moved to electrical sensors like a microswitch and clock (Wilson and Zarrow, 1961) or mercury switches and microcomputers (Buckley et al., 1987). However, limitations persisted, as Wilson and Zarrow (1961) required manual resets at egg collection, and the mercury switches used by Buckley et al. (1987) are no longer available. Many researchers have noted the use of “automatic oviposition recording devices” that measure oviposition within one minute but provide no description of the system or sensors used (Wilson and Sharp, 1975; Wilson and Lacassagne, 1978, Lillpers, 1991).

More recently, Xu et al. (2022) devised a method of utilizing a system with multiple photoelectric sensors in an experimental setting to map where goose eggs were laid in a flat-floor pen. Building on these methods, the objective of this study was to validate the use of a photoelectric sensor to detect the time of oviposition in individually caged broiler breeder hens as a precision tool for research.

## Materials and methods

### Facilities

A 39.2 × 12.5 m solid-sided grow-out facility was used with a negative-pressure ventilation system equipped with exhaust fans, forced-air heaters, cooling pads, and electronic controllers to manage ventilation and temperature. The ambient temperature was set to 21°C. Photoperiod was set to 16 h of light and 8 h of darkness (16L:8D) and light intensity was 40 lux for the entirety of this study.

### Cages

**Fig. 1:**
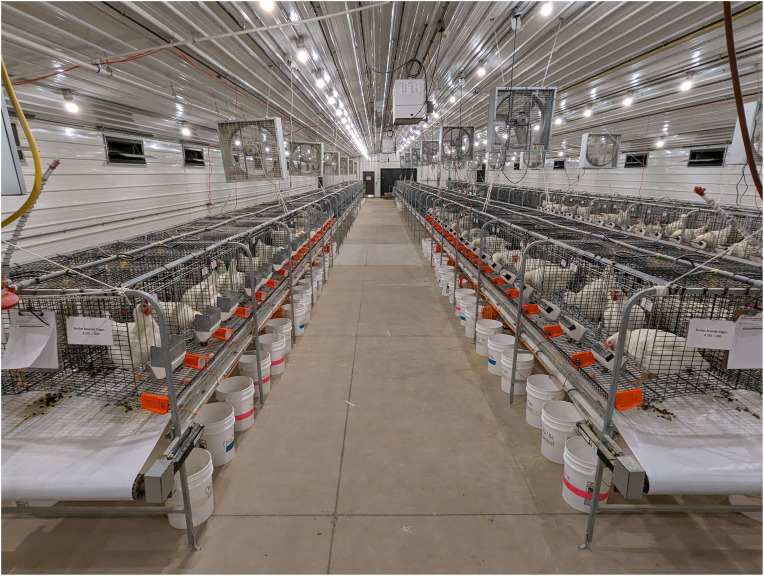
Individually caged broiler breeder hens in six rows of the facility. Photoelectric sensors were installed in the egg saver using an orange 3D-printed divider. (For interpretation of the references to color in this figure legend, the reader is referred to the web version of this article.).

The facility was equipped with six rows of 60 cages (51 × 46 × 61 cm) organized into three banks. Each bank consisted of two opposite-facing rows (Fig. 1). The two rows of cages in each bank shared a manure belt, which was operated each morning. The presence of eggs on the belt beneath each hen was recorded prior to clearing. The cage walls and floor were made of 2.5 × 5.1 cm steel wire mesh (3 mm diameter wire). The front of the cage was comprised of a horizontal sliding wire door with an exterior feeding pan and a 5.7 cm gap at the base to allow eggs to roll into the egg saver. The floor of the cage extended 15 cm to create the egg saver with a 5.7 cm lip. The floor and egg saver (5.1 × 2.5 cm spacing) had a 7 % slope, which was sufficient to roll eggs out of the cage while maintaining leg and footpad health (Tauson, 1998). One nipple drinker provided water to the bird *ad libitum*.

### Animal husbandry

For this validation study, 101 Cobb 500 broiler breeder hens at 42 wks of age were divided across six rows: 20, 15, 21, 16, 15, and 14 birds in rows 1 through 6, respectively. All procedures involving live birds were approved by Auburn University Institutional Animal Care and Use Committee (PRN 2023-5308). Birds received a daily feed allocation at lights-on (7 AM), based on body weight and in accordance with the Cobb Breeder Management Guide (Cobb-Vantress, 2020). Hens were assigned to one of two breeder diets: a standard control diet (2800 kcal) or a high-fiber alternative diet containing 0.8 % Arbocel (2650 kcal). Body weight was recorded weekly, with an average flock body weight of 4.025 kg during this study period with a ∼3 % coefficient of variation (CV). Bird mortality and housing conditions were checked and recorded daily. Total mortality rate was 6.4 % for the duration of the study. Egg collection was completed twice a day in the morning and afternoon. The number of eggs per cage was recorded at each collection, along with any eggs present on the belt beneath the hen. Cracks and soft-shelled eggs were noted. If the egg had not reached the sensors or had been stuck in the cage with the hen and moved by the collector, the time of egg movement was also recorded. Start and end times of collection for each row were recorded to confirm alignment with the sensor records.

### Photoelectric sensor system

***System components.*** For each cage, a polarized retroreflective photoelectric sensor (FMRP-OP-OF, Automation Direct, Cumming, GA) was paired with a reflector (RL116-1, Automation Direct, Cumming, GA) in the egg saver to determine the time of oviposition or egg lay. The photoelectric sensor (**PES**) operates by emitting a visible red light (633 nm) beam towards a reflector, which reflects the light back to the sensor's receiver, creating a closed-loop system. When the beam was complete, the sensor sent a low voltage output (∼8 mV). When an egg rolled into the egg saver and disrupted the light beam, the beam was broken, and the receiver sent a higher voltage output (∼13.5 V).

For each of the three banks of two rows, 16 PES were connected to a datalogger (CR1000X-NA-ST-SW-CC, Campbell Scientific, Logan, UT) and the remainder of PES for each row were connected to an analog input module (Volt116-XD, Campbell Scientific, Logan, UT) using a polyvinyl chloride (PVC) jacketed cable (EVT423, Automation Direct, Cumming, GA) that was suitable for general use and chemical washdown. The datalogger and analog input module were separately housed in a sealed enclosure (NBF-32422, Bud Industries, Willoughby, OH). The logger and analog input module communicated through an ethernet cable.

The logger and PESs were powered with a 12 V power supply (PS200, Campbell Scientific, Logan, UT). Initially, the sensors were powered 24 h each day. After the lights were turned off each day, the team noticed that the red beam from the sensors had an intensity of 35 lux and was enough to photo-stimulate the hens during dark hours. A solid-state relay (D2410, Sensata-Crydom, Attleboro, MA) was used to turn off the sensors when the lighting program turned off the lights.

**Fig. 2:**
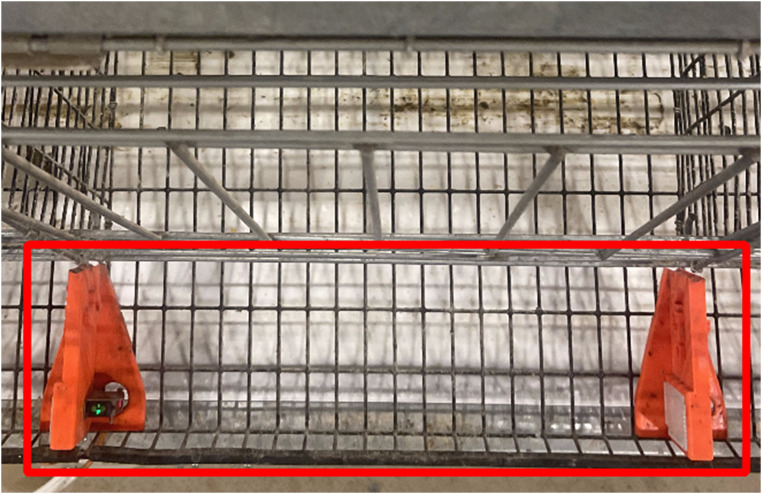
Two 3D-printed dividers were installed in the egg saver of one cage. The PES was located on the right of a divider and the reflector was mounted on the left side of the same divider. The red box in the egg saver highlights the camera detection zone. (For interpretation of the references to color in this figure legend, the reader is referred to the web version of this article.).

***Sensor mount and divider.*** A 3D printed divider was designed to hold the PES mounted on the right side of a divider and a reflector mounted on the left side of the same divider (Fig. 2). The divider replaced the vertical wire dividers in the egg saver between cages. To prevent eggs from getting too close to the reflector or sensor, fenders were added to the base to roll the egg away from either component. Grooves for the steel wire mesh were included in the bottom of the divider for alignment and stability. The dividers were fastened to the egg saver with a bolt and fender washer. The divider was printed on a 3D printer (Prusa i3 MK3S+, Prusa Research, Prague, Czech Republic) with orange filament [Prusament PETG Prusa, Prusa Research, Prague, Czech Republic)].

**Fig. 3:**
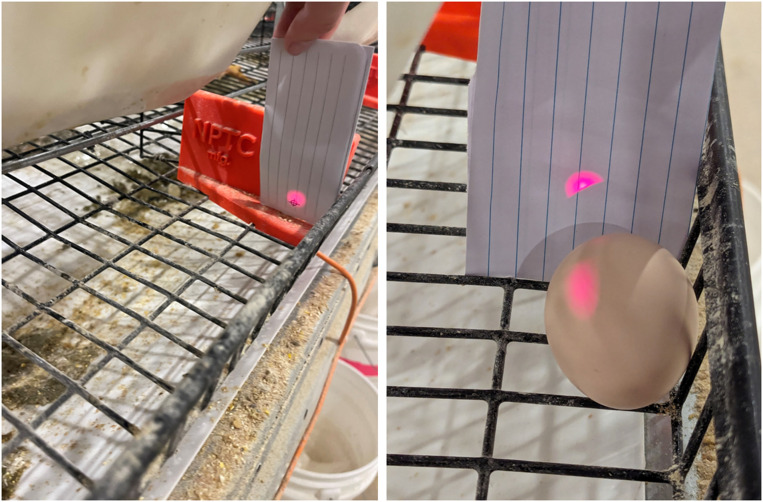
(left) The red beam from the PES light beam hitting a paper card target. (right) The red beam partially missed an egg in the egg saver and did not show the egg present. (For interpretation of the references to color in this figure legend, the reader is referred to the web version of this article.).

The PES was mounted to the divider with a bracket (ST102, Automation Direct, Cumming, GA) that allowed the sensor beam to be adjusted up or down on the reflector plate for fine adjustment. An indicator card was created to provide a target (Fig. 3, left) of where the beam should hit on the reflector plate so that the egg has the best chance of breaking the beam. If not aligned (Fig. 3, right), the egg can roll in the egg saver and not fully break the beam.

### Camera system

**Fig. 4:**
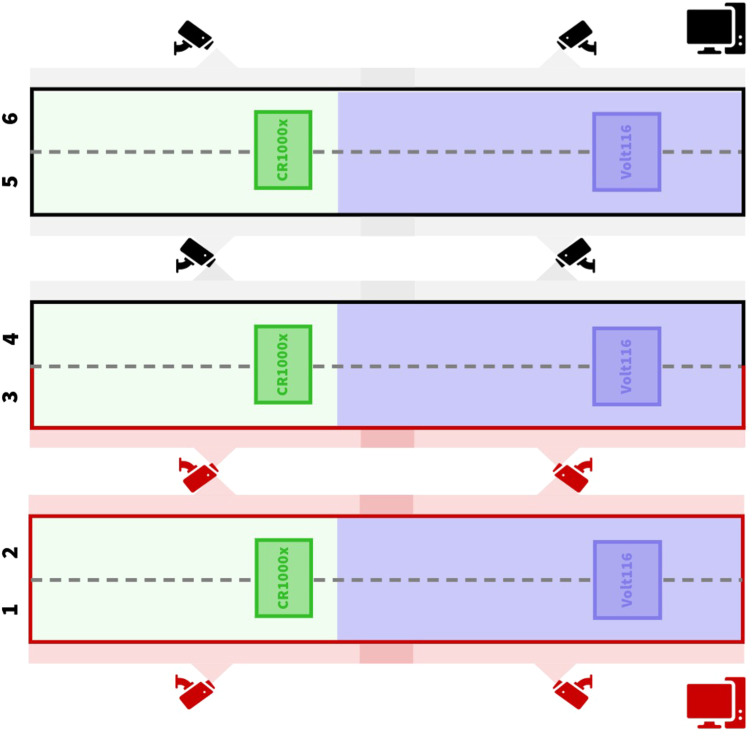
Placement of eight cameras across rows of cages. Two video recording stations were located along the walls.

A camera system was used to validate the time of oviposition determined by the PES system. The system consisted of eight cameras (E841CA-E, Lorex, Linthicum, MD) connected to two network video recording (NVR) systems (N863A64B, Lorex, Linthicum, MD) using ethernet cables. Each NVR with 16 TB of hard drive space (WD85PURZ, Western Digital, San Jose, CA) was housed in an enclosed mobile computer cart and managed with a monitor, keyboard, and mouse. The cameras were mounted to the ceiling with magnets (96650, Harbor Freight, Calabasas, CA) to monitor the egg savers across the six rows (Fig. 4). This arrangement allowed all cages to be monitored with a minimum number of cameras to reduce video storage needs and post-processing efforts.

### Data collection and processing

***Photoelectric sensor system.*** The PES output voltage was monitored during light hours (16 h) by the datalogger at a 1-min sample rate over the two-week period. PES data was downloaded from each system as a text file (.dat).

Data processing was conducted using Excel and RStudio (R-4.4.2). For this analysis, the RStudio packages *dplyr, lubridate,* and *tidyr* were utilized. Raw analog voltage measurements (Table 1) were converted to binary outputs based on a voltage threshold (high voltage (1) = egg present and low voltage (0) = egg not present). Code was developed to search the binary data and identify and count “strings” of consecutive 1′s, which represented when the PES detected an object in front of the sensor. Buckley et al. (1987) noted that their system would detect an oviposition when a hen would peck the sensor and create a false-positive event. They added code to monitor three events to validate that an egg was present in the egg saver and not an artifact from hen movement.

**Table 1:** Example of the data process to determine the time of oviposition.

Time	PES Output	PES Output	String	Notes
	(V)	(Binary)		
12:00	0.0008	0		
12:01	0.0008	0		
12:02	0.0008	0		
12:03	13.5	1	1	Non-egg artifact, bird peck, feather, staff movement. No egg present.
12:04	0.0008	0		
12:05	0.0008	0		
12:06	13.5	1	1	Time of Oviposition: [**12:06**]
12:07	13.5	1	2	
12:08	13.5	1	3	
12:09	13.5	1	4	
12:10	13.5	1	5	The system was looking for the first five consecutive measurements of a broken beam. An egg was present.
12:11	13.5	1		

For each cage, the code started from the first logged event when the lights turned on for each day. Once a 1 was detected, the code counted how many consecutive 1′s followed. In the Table 1 example, the code started counting at 12:03 and then at 12:06. If there were five consecutive 1′s in a string, the system identified an egg that was laid. This egg lay was logged as the timestamp of the first 1 in that string. In the example, the code determined that the time of lay was 12:06. If a string of 1′s occurred immediately when the PES started recording for the day, this string was ignored. This occurred when an egg was laid when the sensors were not active, during the dark period. If no valid string occurred for the day, the PES recorded no egg detected.

***Camera system.*** Overhead cameras recorded video data 24 hr each day, shifting to night vision when dark. Camera data (336 h) was downloaded as (.mp4) in 1-h video packets. Once downloaded as an MP4 file, cages were viewed on-screen and manually recorded for the time of lay. When recording oviposition, the egg had to be in the egg saver bounded by the red box in Fig. 2.

***PES System accuracy.*** Accuracy was determined by comparing the PES system detection time with the camera detection time and staff records over the two-week period. The clocks in the PES systems were synchronized with the clocks in the NVRs and the clocks used by the staff. Table 2 provides definitions for the PES system [detected (D) and non-detected (ND)] and the camera system [observed (O) and non-observed (NO)]. Events were categorized into two successful detection categories (D–O and ND–NO) and two unsuccessful detection categories (ND–O and D–NO) in timing oviposition. The authors set a maximum time difference threshold of 5 min between the PES detected and camera system observed event (D–O) category.

**Table 2:** Oviposition detection event categories.

^1^ The two shaded categories are preferred outcomes when the PES system detections agree with camera and staff observations.

## Results and discussion

### Accuracy

The PES system accurately assessed 1,339/1,414 daily events (101 birds over 14 d) for a total accuracy of 94.7 %. The PES system successfully detected 861 events (D–O; 60.9 %) observed by the camera system and staff records (Table 3). Likewise, the PES system successfully did not detect an egg when an egg was not observed (ND–NO) for 478 events (33.8 %).

**Table 3:** Summary of oviposition detection events for each category.

^1^ The two shaded categories are preferred outcomes when the PES system detections agree with camera and staff observations.

**Fig. 5:**
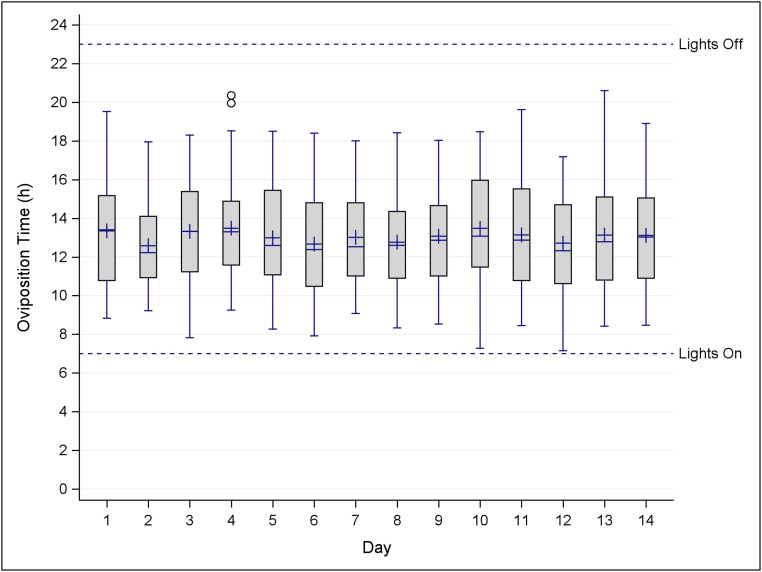
Box-plot of the time of oviposition recorded by the PES system for the 861 events that were both detected and observed (D–O) for all hens over the two-week study period. Mean (+) and median (-) oviposition time is shown for each day. Lights turned on at 7 a.m. and shut off at 11 p.m. each day.

Fig. 5 shows a box plot of the time of oviposition each day recorded by the PES system for the 861 D–O events for all hens. Mean (+) and median (-) oviposition time were shown for all birds each day. The hens roughly laid eggs between 11 AM and 3 PM each day over the two weeks.

### Classification of detection errors

The 14 D–NO and the 61 ND–O events (75 total detection errors; Table 3) were individually classified into categories that could be corrected and those that were uncorrectable. The count of detection errors within each description is summarized in Table 4.

**Table 4:** Classification and count of correctable and uncorrectable detection errors.

	Detection Error Definition	Count	Percent of Total Errors
		(n)	(%)
**Correctable**	Egg picked up by staff outside of PES and camera detection	30	40
	Divider holding sensor or reflector was affected by cage design	15	20
		
		
	Egg was in the egg saver but did not break light beam	9	12
	Egg was in the egg saver but did not break the light beam until bird movement rolled it in later.	3	4
**Uncorrectable**	Egg was laid during dark hours when PES system was off	8	11
	Object other than an egg broke the PES beam	5	7
	Soft shell or cracked egg never rolled into the egg saver	4	5
	Random sensor error	1	1
	**Total Detection Errors**	75	

**Fig. 6:**
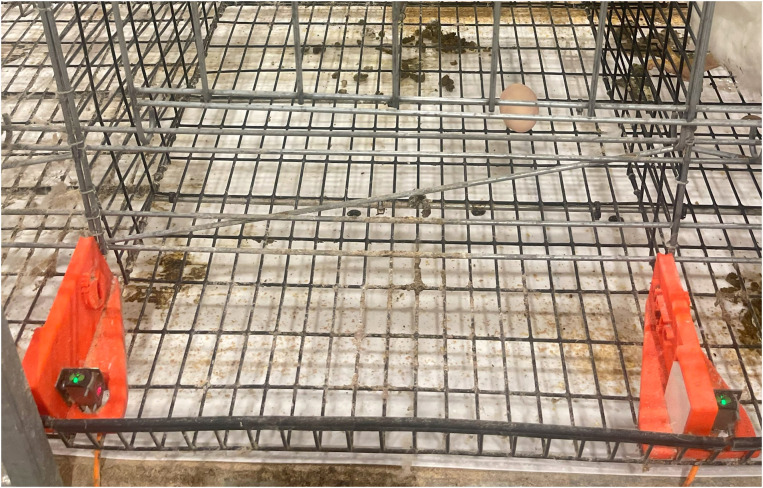
When this egg was laid, it remained in the cage and did not roll into the egg saver.

***Correctable detection errors.*** Of the 75 total detection errors, 57 events can be corrected (Table 4). The largest source of detection errors (30 events) was either the staff recording and picking up the egg, with the egg not breaking the beam, or the camera not showing an egg in the egg saver. The ND–O issue was caused by the steel wire mesh used as flooring inhibiting the egg from rolling into the egg saver and breaking the light beam (Fig. 6).

Eggs not rolling out of the cage was an issue discussed explicitly by past researchers (Weiss and Sturkie, 1952; Wilson and Zarrow, 1961; Buckley et al., 1987). Weiss and Sturkie, (1952) reported that increasing the height of the floor by two inches at the back of the cage mostly reduced these errors, though no estimation of improvement or floor slope was included. During a 400-day study monitoring 110,000 eggs laid, Buckley et al. (1987) reported that 1.9 % of the eggs did not roll far enough to activate the sensors. This error rate was similar to that of this study at 2.1 % (30/1414 events).

**Fig. 7:**
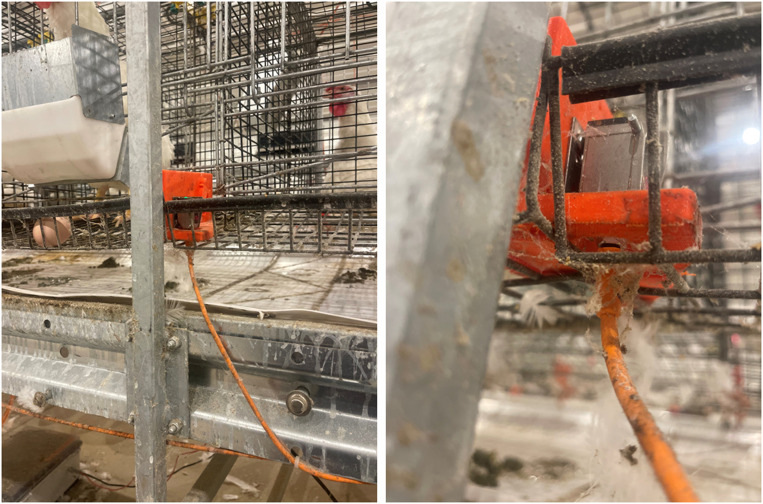
(left) A PES divider mounted where two separate wire mesh panels butt together. (right) A close-up view shows the gap that was created below the divider where the separate wire mesh panels met.

The second largest detection error (15 events) occurred for PES sensors mounted in locations when two separate steel wire mesh panels met (Fig. 7). The panel edges created uneven surfaces for the PES divider to mount on. The separate mesh panels allowed the PES divider to flex and rotate the sensor up and/or down when birds moved in the cage during daily feeding. The misalignment would not reflect the beam back to the receiver.

**Fig. 8:**
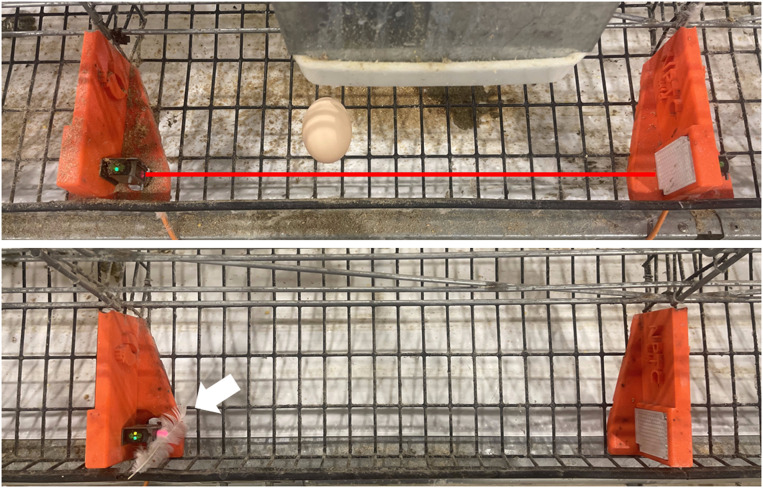
(top) the egg rolled into the egg saver but did not break the PES beam (shown with red line). (bottom) A feather obstructed this sensor showing an egg present. (For interpretation of the references to color in this figure legend, the reader is referred to the web version of this article.).

The last two correctable errors occurred again because of cage design. The first of those (nine ND–O events) was that the egg rolled in the egg saver, and the camera detected the egg. However, the egg did not roll all the way to break the light beam (Fig. 8 top). The last correctable error (three events) was because the PES detected the egg, but the time stamp was greater than 5 minutes after the camera detected the egg. Again, due to the flooring, there was a lag created because the egg was slow to roll. Movement of the hen in the cage caused the egg to roll later than the 5-min window.

Most of the correctable detection errors may be resolved by improving the cage floor design to improve the egg rolling into the egg saver. Improvements could include material with a smaller mesh pattern and/or a thicker gauge wire to keep the floor flat. Increasing the floor slope steeper than 7° may help the egg roll more easily. Mather et al., (1965) reported that increasing the floor slope to greater than 15° would ensure that most normal-shelled eggs would roll properly. However, this far exceeds the 8° maximum slope regulated by United Egg Producers (United Egg Producers, 2017), aimed to balance egg quality with the health of hens (Tauson, 1998).

The remaining errors were due to staff picking up eggs outside the detection area or rolling the egg into the beam but only leaving it for a few seconds. The authors have developed improved protocols for staff to roll the eggs into the light beam and leave them for at least five minutes. These improvements can improve the PES system detection accuracy to 98.7 %.

***Non-correctable detection errors.*** Of the 75 total detection errors, 18 events could not be corrected (Table 4). The PES system was turned off when the lights were turned off to prevent photorefractoriness of the hens when they are provided exposure to low intensity red light beyond their 16 h photoperiod (Hanlon et al., 2023). A total of eight eggs were not detected but observed (ND–O) with cameras when they were laid in the dark.

The PES detected another five events (0.3 %) when an egg was not observed (D–NO). These events were created when objects like feathers broke the beam (Fig. 8 bottom).

Staff records and cameras observed four soft-shell or cracked eggs that the PES did not detect (ND–O). While these eggs would not be counted as settable fertile eggs, the PES system would not be able to note the time of oviposition for these eggs in a research setting. Staff records could note the soft-shell eggs external to the PES system. Mather et al. (1965) reported that 5 % of the eggs not detected in their study were due to soft-shell and malformed eggs that failed to roll and activate the sensor. One event was detected by the PES with no written record (D–NO), camera observation, or visible obstruction on the sensor. This detection error was labeled a random error.

Table 5 compares this study (94.7 % detection accuracy) to past studies using various methods to record the time of oviposition. Warren and Scott (1936) reported 100 % detection accuracy for 40 individually caged hens using staff monitoring every hour between 6 am and 5 pm. As discussed, this method is tedious and can become impractical as more birds are used in an experiment. Buckley et al. (1987) monitored 576 individual birds with 98.1 % detection accuracy using mercury switches and a microcomputer. Mercury switches are no longer available. Xu et al. (2022) tested multiple photometric sensor heights and sensor distances from the egg to determine the floor position location and found that accuracy was better than 87.5 %.

**Table 5:** Comparison of the photoelectric sensor (PES) system to past research methods for detecting the timing of oviposition in caged chickens and geese.

Study	Detection System	Recording Method	Cages Used	Floor Slope	Time Resolution	Detection Accuracy	Notes
			(n)			(%)	
Warren and Scott (1936)	Physical Monitoring	Paper	30 - 40		1 h (6am – 5pm)	100	-
Hinds (1949)	Spring Hinged Lever with Contact Switch	Paper: Kymograph Drum	24	30°	3 h	N.R.	Drum needs daily reset/cleaning
Weiss and Sturkie (1952)	Copper Mesh Circuit and Stylus	Paper: Kymograph Drum	18	2-in drop	< 15 min	N.R.	Drum needs daily reset/cleaning
Bastian and Zarrow (1954)	Stylus	Kymograph Drum	24	N.R.	15 min	N.R.	Drum needs daily reset/cleaning
Wilson and Zarrow (1961)	Microswitch with Trip Wire	Clock	> 24	N.R.	1 min	N.R.	Clock needs to be reset after each oviposition
Mather et al. (1965)	Mercury Switch with Swinging Gate	Mechanical rotary switch and recorder	≤ 133	>15°	20 min	95	-
Lowe et al. (1985)	Pressure plate and microswitch on egg belt	Computer	20	N.R.	variable	N.R.	Experimental prototype, not validated.
Buckley et al. (1987)	Mercury Switch with Swinging Gate	Microcomputer	576 - 1024	11°	2.4 to 4.2 min	98.1	Mercury switch has potential to break and release Mercury
Xu et al. (2022)	Photoelectric Sensor	Computer	1	N.A.	3 sec	≥ 87.5	Floor pen trial looking for goose egg location
This study	Photoelectric Sensor	Datalogger	101	7°	< 3 min	≥ 94.7	Can only be used during light hours

Other than staff visually monitoring cages, every system described some set of cleaning, adjustment, and/or maintenance activities required to operate the systems accurately. Beyond adjusting a few PES sensors to compensate for the cage flooring design issues in this study, the PES system recorded oviposition daily with little effort from the team. For longer duration studies, it would be good to have a protocol for staff to wipe any heavy dust buildup from the PES sensor face and the reflector face.

### Detection resolution

Within the 861 D–O events, the time difference between the PES detection and camera detection was calculated. All PES detection events were recorded less than three minutes from the camera system observation. The PES detections were within one minute of the camera observation for 20.7 % of events, between one and two minutes for 74.8 %, and between two and three minutes for 4.5 % of events.

Most studies in the past with staff manually monitoring oviposition have operated on a one-hour resolution for practical purposes (Warren and Scott, 1936; Giesen et al., 1980; Fasenko et al., 1992; Lewis et al., 2004a; Zakaria et al., 2005, 2009; Akil and Zakaria, 2015). Table 5 compares the time resolution in this study to past studies using systems to monitor oviposition. Hinds (1949), Weiss and Sturkie (1952), and Bastian and Zarrow (1954) used a lever or stylus connected to a kymograph drum to mechanically record oviposition with a 15-min resolution. Mather et al. (1965) had a 20-min resolution when using a mechanical rotary switch and recorder.

Wilson and Zarrow (1961) used a trip wire and an electric clock to record the time of oviposition with a one-min resolution. The clocks had to be manually reset each day. Buckley et al. (1987) had a similar detection resolution to this study between 2.4 and 4.2 min. This resolution was driven by the time it took the microcomputer to poll each of the 576 or 1024 cages, respectively. These past studies reported similar challenges to our study in getting the egg to roll into the sensing area. Unlike our study, none of the studies reported visual monitoring to assess the time between when the egg was laid in the cage and when the egg was recorded with each system.

### Labor

The initial installation of the PES systems was approximately 74 hours of labor, with half dedicated to fabricating the systems and the other half to developing the software code and installing the sensors on the cages (Table 6). Downloading and processing the data during the two-week study took approximately 10 minutes. The initial installation of the camera systems took less than 10 hours to set up, significantly less than the PES systems (Table 6). However, video downloading, processing, and cross-validation with staff records totaled 109 hours. Using the PES system beyond this two-week study would only require time for downloading and processing at 10-min per two-week period. Whereas the time to process the videos would increase significantly (30 wk study would require 1,500+ hrs to process). The PES system really shines as it allows users to download and evaluate data as the study progresses, whereas processing video is a slow process.

**Table 6:** Comparison of labor required for the photoelectric sensor (PES) and camera systems.

	Study Phase	Labor Category	Time	Subtotal	Total
			(Hours)	(Hours)	(Hours)
**PES System**	**Initial Install**	Fabrication of PES Systems	35.0	73.8	74
		Installation on Cages	10.5		
		Datalogger Programming	0.3		
		Initial Code Script	28.0		
	**Data Processing**	Downloading Data	0.1	0.02	
		Pre-R Data Processing	0.1		
		Code Execution	0.015		
**Camera System**	**Initial Install**	Fabrication of Camera Systems	2.0	9.3	118
		Installation of Cameras	7.0		
		Camera Settings Setup	0.3		
	**Data Processing**	Downloading Footage	36.0	108.7	
		Cage Labeling for all Cameras	2.7		
		Looking for egg lay and cross-validation with staff records	70.0		

### Data storage

For the two-week period, approximately 4,032 gigabytes (GB) of storage were required to store video data for analysis. In contrast, only 16 megabytes of storage were required to store data from the PES system for analysis. Moving to a year-long study would require 106 terabytes for video storage compared to the 0.5 GB needed for the PES system.

### Costs

There are a variety of photoelectric sensors and datalogging systems that can be used to create the PES system. However, due to the dirty and wet environment, the authors chose industrial-grade sensors and dataloggers used in challenging environments. The total cost of the PES system was $35,000 plus $2,220 in labor ($369 per cage. Though this may seem to be a high unit cost, we had no issues with sensors not working or lost data due to power losses or system glitches with the PES system.

The camera systems have worked reliably in past poultry research environments, with failures only occurring if the electricity to the building went out. With only eight cameras and two NVRs, the system costs were $3700 plus $3,570 in labor ($72 per cage). Though cameras were only 20 % of the costs of the PES system, the labor and speed of analysis would be worse as the number of cages increased.

## Conclusions

A PES system was developed to determine the time of oviposition in individually caged broiler breeder hens. The PES system accurately detected 1339/1414 events (94.7 %) within 3 min of the camera observation. Addressing correctable errors caused by cage design issues could improve the system accuracy to 98.7 % (1396/1414). Current limitations of the PES system include the inability to detect soft-shelled or cracked eggs that do not roll into the egg saver, the inability to distinguish when a hen has laid two eggs in the egg saver for a collection period, and the requirement to deactivate the system during the dark period to prevent photorefractoriness of broiler breeders by the red light emitted from the sensors. Post-processing and determination of the time of oviposition was simplified and more efficient using a PES system (< 0.2 h) compared to a camera system (>100 h) for a 14-d data set. At an estimated cost of $369 per cage, the PES system offers a reliable, high-precision tool to explore any poultry egg production research where non-invasive identification of oviposition timing could provide insight into reproductive capacity and success.

## CRediT authorship contribution statement

**L.R. Sroda:** Writing – original draft, Validation, Methodology, Formal analysis, Data curation. **J.D. Davis:** Writing – review & editing, Writing – original draft, Project administration, Methodology, Formal analysis, Data curation, Conceptualization. **K. Diehl:** Writing – review & editing, Methodology, Conceptualization. **C.M. Edge:** Writing – review & editing, Methodology. **M.R. Berger:** Writing – review & editing, Validation, Supervision, Methodology. **K.E.C. Elliott:** Writing – review & editing, Resources. **B.R. Flack:** Writing – review & editing, Funding acquisition. **C.L. Hanlon:** Writing – original draft, Funding acquisition, Data curation, Conceptualization.

## Disclosures

The authors of this manuscript have no conflicts of interest with the paper submitted. The project was funded in part by a Cobb Research Initiative and by a USDA project. Authors' time was supported by the USDA project and institutional funding sources as described in the paper acknowledgements.
